# Investigating the Secreted Proteome of Primary and Metastatic Human Brain Tumour Explants Maintained on a Miniaturised Perfusion Device

**DOI:** 10.3390/curroncol33040182

**Published:** 2026-03-25

**Authors:** Samuel G. Perkins, Sabrina F. Samuel, Richard J. Digby, Heiko Wurdak, John Greenman, Ryan K. Mathew

**Affiliations:** 1 School of Medicine, University of Leeds, Leeds LS2 9JT, UK; samuel.perkins2@nhs.net (S.G.P.); s.f.samuel@leeds.ac.uk (S.F.S.); richard.digby1@nhs.net (R.J.D.); h.wurdak@leeds.ac.uk (H.W.); 2 Department of Neurosurgery, Leeds General Infirmary, Leeds Teaching Hospitals NHS Trust, Leeds LS1 3EX, UK; 3 Department of Cellular Pathology, St. James’s University Hospital, Leeds Teaching Hospitals NHS Trust, Leeds LS9 7TF, UK; 4 Centre for Biomedicine, Hull York Medical School, University of Hull, York YO10 5DD, UK

**Keywords:** biomarker, low grade glioma, glioblastoma, brain metastasis, tissue perfusion device, mass spectrometry, early diagnosis

## Abstract

Background: Non-invasive approaches to brain tumour detection and diagnosis are limited by the absence of clinically validated circulating biomarkers. This study utilised a miniaturised tissue perfusion model to maintain human brain tumour tissue ex vivo with the aim of identifying tissue-derived proteins with potential biomarker utility. Methods: 55 tumour samples from 11 different brain tumours (glioblastoma *n* = 4, low-grade glioma *n* = 4, brain metastases *n* = 3) were micro-dissected and maintained ex vivo on a continuous-flow perfusion device for 168 h. Proteomic analysis of tumour effluent was performed by reversed-phase capillary liquid chromatography-mass spectrometry. Two candidate proteins—extracellular matrix protein 1 (ECM1) and cathepsin D—were quantified using ELISA. Results: All tumour subtypes retained tissue viability over 168 h of perfusion. Proteomic profiling identified 90 tissue-derived proteins in the tumour effluent. Many proteins corresponded to previously described cancer biomarkers such as glial fibrillary acidic protein (GFAP) while others, including Serpin A12 and collapsin response mediator protein-2 (CRMP2), had not yet been described in a brain tumour context. ELISA confirmed significantly higher ECM1 levels in high-grade glioma effluent compared with low-grade glioma (*p* = 0.0407), whereas cathepsin D levels did not differ significantly between tumour types. Conclusions: The ex vivo perfusion model effectively preserved primary and metastatic human brain tumour tissue and enabled direct characterisation of tumour-secreted proteins. The proteins identified here warrant further validation as tumour biomarkers in patient serum or cerebrospinal fluid.

## Simple Summary

Brain tumours are relatively rare and particularly difficult to diagnose because simple diagnostic tests such as blood tests are not yet in routine clinical practice. Typically, expensive and lengthy brain scans are required. In this study, we kept human brain tumour samples alive outside the body using a microfluidic device to supply the tissue continuously with nutrients whilst simultaneously removing waste products. This allowed us to directly study the proteins released by the tumours, with the goal of identifying those that could form the basis of a diagnostic blood test. We detected many proteins that have previously been reported as potential tumour markers, as well as several that have not been studied in this context before. Some of the proteins identified were linked to tumour aggressiveness. The combination of tissue-based microfluidics and proteomic analysis have provided a novel way of determining blood-based markers for early and accurate brain tumour detection and diagnosis.

## 1. Introduction

Brain tumours are a heterogenous group of benign and malignant pathologies that frequently prove challenging in diagnosis as well as treatment. Gliomas are the most common primary tumour of the central nervous system (CNS) and are classified by the World Health Organisation (WHO) as grades 1–4, reflecting a spectrum of low to high malignancy [1]. Adult-type diffuse gliomas are classified on the basis of molecular characteristics including isocitrate dehydrogenase (IDH1/2) mutation and 1p/19q codeletion status, whilst the grade of tumour is determined by a combination of histopathologic and molecular analysis. Glioblastoma (GBM) is the most prevalent and malignant glioma subtype (WHO grade 4), defined molecularly by wild-type IDH status. GBM exhibits rapid proliferation, treatment resistance, and a five-year survival rate of approximately 5% [2], one of the lowest among all human cancers. In contrast, astrocytomas (WHO grades 2–4) and oligodendrogliomas (WHO grades 2–3) are IDH-mutant and display more variable clinical behaviour. Lower-grade gliomas (WHO grades 1–2) typically demonstrate a low proliferative index but may still cause significant morbidity and carry a substantial risk of malignant transformation that is estimated to occur in 23–72% of cases, with a median time to transformation of between 2.7 and 5.4 years [3].

Primary brain tumours comprise only a subset of the brain tumours encountered in clinical practice. The metastasis of extra-cranial malignancies to the brain is common, occurring in 20% of all cancer patients. It is estimated that 75% of malignant brain tumours are metastases, most frequently arising from lung, breast, colorectal, or melanoma primaries [4,5]. The spread of cancer to the brain is often associated with poor disease outcomes and, in many cases, significant neurological impairment. Although brain metastases typically arise in the context of established disseminated disease, the recognition of brain metastases can be the index event in the diagnosis of a patient with cancer (termed a Cancer of Unknown Primary; CUP)—as is particularly common with melanoma [6].

For some patients, the diagnosis of a brain tumour follows the onset of neurological symptoms, whilst in others the diagnosis is incidental, occurring during investigation for unrelated symptoms (e.g., a head injury). In both instances it is typically the recognition of abnormalities on brain imaging that alert clinicians to the possibility of a brain tumour. Modern magnetic resonance imaging (MRI) techniques such as diffusion-weighted imaging and spectroscopy allow for detailed characterisation of suspicious lesions, but, despite these advances, tissue biopsy is required to confirm the type and grade of tumour and to provide material for molecular analysis. Surgical tissue biopsy is an invasive procedure that carries a risk of damage to brain structures. In some circumstances tumour location may render the procedure infeasible. Given the grave prognosis conferred by GBM there is a clear need for early and accurate diagnosis to facilitate timely intervention and care planning.

To complement radiological and histopathological approaches to diagnosis, much attention has turned to identifying serum or cerebrospinal fluid (CSF) brain tumour biomarkers (liquid biomarkers). Investigated analytes include circulating tumour DNA (ctDNA), circulating tumour cells (CTCs), extracellular vesicles (EVs), and soluble proteins. None have yet entered clinical practice, but several promising candidates have emerged. Glial fibrillary astrocytic protein (GFAP) is among the most extensively studied. Serum GFAP is detectable in 62.7% of high-grade glioma patients [7] and correlates with tumour volume and necrosis. However, variable tumour expression and elevations in non-neoplastic CNS lesions limit its diagnostic sensitivity and specificity [8]. Other proteins, including S100 family members [9], vimentin [10], and YKL-40 [11], have also shown potential but only in small cohorts. It is possible that panels of multiple serum proteins may be able to provide greater diagnostic accuracy than a single biomarker, as indicated by Nijaguna et al. (2015) who reported an 18-cytokine signature in serum able to discriminate glioma patients from healthy individuals with 96.55% accuracy [12].

The sequencing of ctDNA presents an alternative approach to tumour diagnosis and prognostication that provides insight into tumour genetics and allows the identification of target mutations such as IDH1 R132H [13]. In patients with brain tumours, the levels of ctDNA in the plasma are low and inconsistently detectable—presumably a consequence of the blood–brain barrier (BBB). Approaches that profile CSF have shown relative enrichment of tumour-derived DNA, allowing for more reliable detection of target mutations [14].

EVs derived from brain tumours carry DNA, RNA, proteins and lipids which together reflect the identity of their malignant cell-of-origin. An EV-derived panel of seven-miRNAs has been shown to distinguish GBM patients from controls with high specificity [15]. EV-derived miR-21 has also been shown to differentiate gliomas from controls, but not high-grade gliomas from brain metastases [16]. The implementation of larger scale trials on the diagnostic utility of EVs is warranted, but efforts are limited by a lack of standardised and reproducible methods of isolating these microparticles. Free-circulating miRNAs present an alternative means of glioma diagnosis. A 2018 meta-analysis of 16 publications involving 2325 participants reported the diagnostic specificity and sensitivity of circulating miRNAs (specificity 0.87 and sensitivity 0.86) as similar to that of proton magnetic resonance spectroscopy (specificity 0.87 and sensitivity 0.86). The authors did however report large heterogeneity amongst the included studies and advocated for the use of standardized sample processing and miRNA detection methods before introduction into clinical practice can be considered [17].

Additional research that demonstrates the specificity and utility of liquid biopsies in large real-world cohorts is necessary before their adoption into routine clinical practice. The prospective EMBRACE study is designed to evaluate the clinical performance of a spectroscopic liquid biopsy platform (Dxcover^®^) for early brain tumour detection. The trial intends to recruit approximately 2200 patients presenting to primary care with non-specific symptoms that may be suggestive of a brain tumour—outcome data are yet to be reported [18,19]. Similarly, a blood test for glial tumours based on the detection of circulating glial cells (TruBlood^®^) has undergone recent evaluation in four clinical observational studies [20]. Published data report high analytical sensitivity (95%) and specificity (100%), suggesting a potential role for this technology in supporting glioma diagnosis in future. The test, in its current iteration, however, is not intended to provide information on tumour grade or subtype.

Current glioma diagnosis still relies heavily on molecular markers from tumour tissue obtained via biopsy with no serum or CSF markers having reached clinical utility [21]. The low concentration of these markers in serum, practical limitations to CSF sampling, and a lack of standardised methodologies limits the implementation of large, well-powered studies. In addition, it is important that prospective markers are evaluated for the specificity by which they differentiate primary brain tumours from metastases, however, this is infrequently addressed in the existing body of research. It is recognised that biomarkers reported in plasma may reflect local or systemic responses to brain tumourigenesis. Whilst such markers may accurately differentiate brain tumour patients from controls, the specificity by which they can distinguish between tumour subtypes is likely to be lower than that of markers which are directly released from tumour tissue [22].

In the search for novel tumour biomarkers, many studies have profiled the serum or CSF proteome. However, the high background protein content of these biological fluids poses significant challenges for the detection of proteins released at sub-nanomolar concentrations during brain tumourigenesis [23,24]. These approaches also cannot distinguish proteins released by tumour cells from those released in a reactive manner from non-malignant tissues. To address these limitations, we have taken the novel approach of directly interrogating the secreted proteome of brain tumour explants maintained in an ex vivo miniaturised perfusion system. Our group has recently demonstrated that human GBM tissue can be maintained successfully ex vivo using a system that enables the continuous flow of nutrients and the removal of waste, whilst preserving tumour morphology and microenvironment [25,26,27]. The utility of a similar model has been demonstrated with thyroid cancer, with which novel biomarkers were identified in the tissue effluent of thyroid tumour explants [28,29].

In the present study we demonstrate the successful maintenance of multiple types and grades of brain tumour in an ex vivo miniaturised perfusion system. Using reverse-phase capillary liquid chromatography-mass spectrometry, we identified several proteins in the tumour tissue effluent that have previously demonstrated potential diagnostic utility in glioma. In addition, we identified multiple proteins not yet studied in the context of biomarker research, which we propose as novel candidates for further investigation with potential to advance the development of non-invasive brain tumour diagnostics. Two of these, cathepsin D and extracellular matrix protein 1 (ECM1), were further investigated by ELISA to verify the mass spectrometry data and measure changes over time.

## 2. Materials and Methods

### 2.1. Patient Sample Acquisition

The project was conducted under the ethical approval of Leeds NHS Research Ethics Service via Neuropathology Research Tissue Bank (20-YH-0109; 21 May 2020). Patients over 16 years of age undergoing diagnostic or therapeutic resection of a primary or secondary brain tumour at Leeds Teaching Hospitals NHS Trust provided consent for inclusion in the study. Recruitment took place between May and December 2023. Participants lacking the capacity to provide informed consent were excluded. Only surplus tumour tissue not required for clinical histopathological analysis was used in this study.

### 2.2. Perfusion Device Configuration

**Figure 1:**
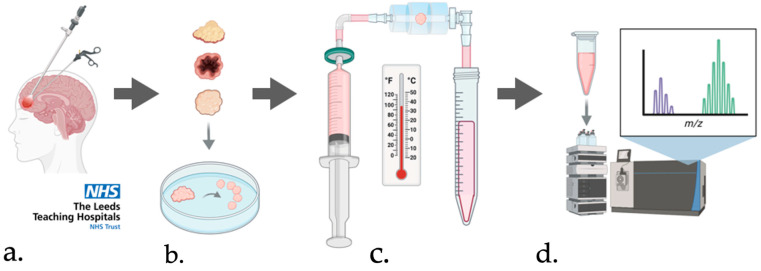
Visual summary of the sample acquisition and testing process: (**a**) surgical acquisition of tumour tissues; (**b**) microdissection of tumour tissue into 25 mg ± 10% samples; (**c**) maintenance of tumour samples within the miniaturised perfusion device at 37 °C; (**d**) analysis of the tissue effluent by reversed-phase capillary liquid chromatography-mass spectrometry. Created in https://BioRender.com.

The perfusion device comprised a custom laser-cut (LS6840, HPC Laser, Halifax, UK) polymethyl methacrylate flow cell, attached via 1/32″ Tygon silicone tubing (Cole-Parmer, Vernon Hills, IL, USA) to a 0.22 μm-filtered 20 mL syringe containing culture media. The flow cell inlet and outlet were 4 mm in diameter, to enable direct interfacing with Luer-to-barb tubing connectors. The internal chamber, also 4 mm in diameter, housed the tissue sample which was held in place by a retaining layer perforated with 37 × 100 μm holes to allow the through-flow of media. Media was perfused through the device at a flow rate of 2–3 μL min^−1^ via a PHD-ULTRA syringe pump (Harvard Apparatus, Holliston, MA, USA). The outlet was connected via the same tubing to allow collection of the effluent media in 15 mL centrifuge tubes [27]. A schematic showing the key points in the process is shown in Figure 1.

### 2.3. Micro-Dissection and Perfusion

Following surgical excision, brain tumour biopsy tissue was transported to the laboratory on ice and processed within 2 h. The tissue was washed in phosphate-buffered saline (PBS) and processed using single-use sterile curved blade no. 2 scalpels (Swann–Morton, Sheffield, UK) and forceps. The biopsies were micro-dissected and weighed into five 25 mg ± 10% samples, with efforts taken to exclude areas of necrosis or cauterised tissue. The five samples were then placed in the internal chamber of the perfusion device (one tissue sample per device), which had been sterilised with 5% (*w*/*v*) Chemgene/70% (*v*/*v*) ethanol, then extensively flushed with sterile media (the same media used for perfusion) to remove disinfectant and ensure no air was trapped in the apparatus. The tissue-containing devices were transferred to an incubator set to 37 °C, where they were perfused simultaneously with media for a period of 168 h (7 days). The centrifuge tubes receiving the tissue effluent were replaced each 24-h period, and the effluent was stored at 4 °C for up to 24 h prior to aliquoting and transfer to −20 °C. In addition to the samples maintained on the perfusion device, a portion of fresh tumour tissue taken at the start was fixed in 4% (*w*/*v*) paraformaldehyde for histological analysis.

### 2.4. Tissue Viability Assay

The composition of the perfusion media was dependent on the tumour type. The decision regarding the composition of the media was made with consideration of previously described tissue culture protocols [30,31,32]. Foetal bovine serum was omitted from the media to facilitate proteomic analysis of the effluent. The composition of the media is detailed in Appendix A Table A1. Tissue viability was assessed using a colorimetric lactate dehydrogenase (LDH) assay with the Cytotoxicity Detection Kit (Roche, Basel, Switzerland). The LDH assay was performed as per the manufacturer’s protocol in triplicate on effluent collected at each 24-h time point. Absorbance was recorded at 495 nm using Berthold Mithras LB 940 (Berthold Technologies GmbH & Co. KG, Bad Wildbad, Germany). The background absorbance of the pre-perfusion media was subtracted from the absorbance of tissue effluent (post-perfusion media), and the average absorbance for each sample was calculated and normalised per milligram of tissue weight.

### 2.5. Histology

Histological analysis was performed on tissue after 7 days of maintenance on the perfusion device, as well as on fresh post-operative tissue. Tissue was fixed in 4% (*w*/*v*) paraformaldehyde followed by 30% (*w*/*v*) sucrose solution before being frozen in optimal cutting temperature medium (OCT) (Avantor, Radnor, PA, USA) and stored at −80 °C. Tissue was sectioned (8 µm) using a cryostat microtome and stained with haematoxylin and eosin. Images were acquired using Axioscan Z1 Slidescanner (Carl Zeiss Microscopy GmbH, Oberkochen, Germany) and analysed using QuPath (version 0.5.1) [33]. Images were assessed and reported by a qualified neuropathologist.

### 2.6. Proteomic Analysis

Effluent from each tumour at the 120-h time point was used for proteomic analysis by reversed-phase capillary liquid chromatography-mass spectrometry (RP-cLC-MS). This time point was chosen in view of early data which showed that cell death on the perfusion device stabilised by 120 h [25]. The perfusion media was analysed in the same manner as the effluent to provide the control values and allow identification of peptides unique to the tumour effluent. Proteomic sample processing was completed as follows: 8 μL of the effluent media was fractionated in two fractions—albumin-depleted and albumin-rich—and processed by tryptic digestion as previously described [34]. Peptides were separated online by reversed-phase capillary liquid chromatography on an EASY-nLC 1000 UPLC system connected to a 20-cm capillary emitter column (inner diameter 75 μm, 3 μm Reprosil-Pur 120 C18 media). The UPLC was connected to an Orbitrap Exploris 240 mass spectrometer (Thermo Fisher Scientific, Waltham, MA, USA). The acquisition time was 45 min. The main chromatographic gradient was 4–25% (*v*/*v*) acetonitrile in 0.1% (*v*/*v*) formic acid. The MS scan resolution was set to 60,000. Up to 20 most intense multiply charged ions per scan were fragmented in the MS2 mode, with the MS2 resolution set to 15,000. Data were processed against Uniprot human protein sequence (January 2024) and Uniprot bovine protein sequence (January 2024) databases by MaxQuant 1.6.3.4 software [35]. Carbamidomethylation of cysteine was set as a fixed modification. N-terminal protein acetylation, oxidation of methionine, and deamidation of asparagine and glutamine were set as variable modifications. At least one unique peptide for valid protein identification was selected.

### 2.7. Quantification of Biomarker Release Using ELISA

Two of the proteins identified by mass spectrometry—cathepsin D and ECM1—were chosen for further quantitative evaluation using ELISA (Human ECM1 DuoSet DY3937; Human Cathepsin D DuoSet DY1014) (Bio-Techne, Minneapolis, MN, USA). Both ELISA evaluations were carried out according to the manufacturer’s instructions. The lowest level of detection was 15.6 pg/mL for ECM1 and 62.5 pg/mL for cathepsin D. The concentration of both proteins in the tissue effluent was assessed over different time points and compared between different tumour types. Due to the availability of tissue effluent, the comparison between tumour types was performed with HGG effluent collected at 72 h and with LGG and brain metastasis effluent collected at 96 h.

### 2.8. Statistical Analysis

Data were grouped as low-grade glioma (n = 4), high-grade glioma (n = 4) and brain metastases (n = 3), and were analysed using one-way ANOVA followed by Tukey’s multiple pairwise comparison test. Residuals were visually inspected for normality using Q–Q plots. Statistical analysis was carried out using GraphPad Prism (GraphPad Software version 10.0). *p* values ≤ 0.05 were considered significant. All graphs display mean ± standard error of the mean (SEM) unless otherwise specified.

## 3. Results

### 3.1. Demographics

Eleven human brain tumour specimens were utilised in the study, each was processed to yield five samples per tumour. A total of 55 tumour samples were then maintained on the miniaturised perfusion device. Of the 11 tumours there were four GBM, four LGG and three brain metastases. The demographics and clinical characteristics of the 11 patients are summarised in Table 1.

### 3.2. Histopathology

**Figure 2:**
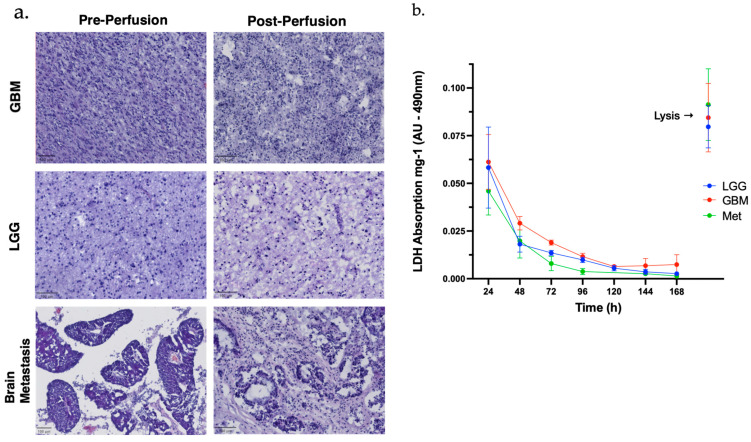
(**a**) Representative H&E images of glioblastoma (GBM), astrocytoma (LGG) and colorectal adenocarcinoma brain metastasis pre- (left) and post- (right) maintenance on the tissue perfusion device; (**b**) average LDH absorbance from the effluent media collected at 24-h intervals over a 168-h time period. The average value ± SEM for each tumour type is plotted. The viability of the tissue at 168 h is demonstrated by the peak in LDH signal, taken to reflect lysis of residual viable tissue, observed after the addition of lysis solution.

The impact of 168 h of ex vivo perfusion on the tumour tissue architecture was evaluated by histopathology. H&E-stained sections from tissue taken prior to and following perfusion were assessed by a neuropathologist. In the LGG samples, overall tissue viability was maintained with high cellularity observed both pre- and post-perfusion. A subset of specimens demonstrated increased friability, potentially attributable to processing artefact or degradation of the background neuropil matrix during perfusion. The GBM specimens exhibited marked heterogeneity with variable cellularity. Foci of necrosis were evident in several pre- and post-perfusion samples. Despite these features, general tissue preservation was achieved, although a reduction in cellular density was noted in some regions post-perfusion. Cytomorphological characteristics remained readily identifiable (Figure 2a). Among the brain metastases, the metastatic lung adenocarcinoma retained cytomorphological integrity; however, the glandular architecture was less readily appreciable post-perfusion. In contrast, the metastatic colorectal adenocarcinoma demonstrated partial preservation of glandular architecture post-perfusion (Figure 2a). The metastatic breast carcinoma was notably well-preserved post-perfusion, with all assessed samples demonstrating abundant viable tumour cells and minimal evidence of structural degradation showing patient to patient variability.

### 3.3. LDH Absorbance

The release of LDH into the effluent media, as measured by formazan dye production, followed a similar pattern across all samples. There was an initial peak in LDH release, attributed to tissue damage during the microdissection process, followed by a decline to a steady state of LDH release (Figure 2b). The average pooled LDH absorbance for each tumour type did not differ significantly between tumour types at each time point. At the 168-h time point one tissue sample from each tumour was removed from the perfusion device and transferred to a lysis solution. Lysis of the tissue was associated with an average 37.16-fold increase in LDH, which was taken to reflect the release of LDH from residual viable tissue (Figure 2b).

### 3.4. Mass Spectrometry

**Figure 3:**
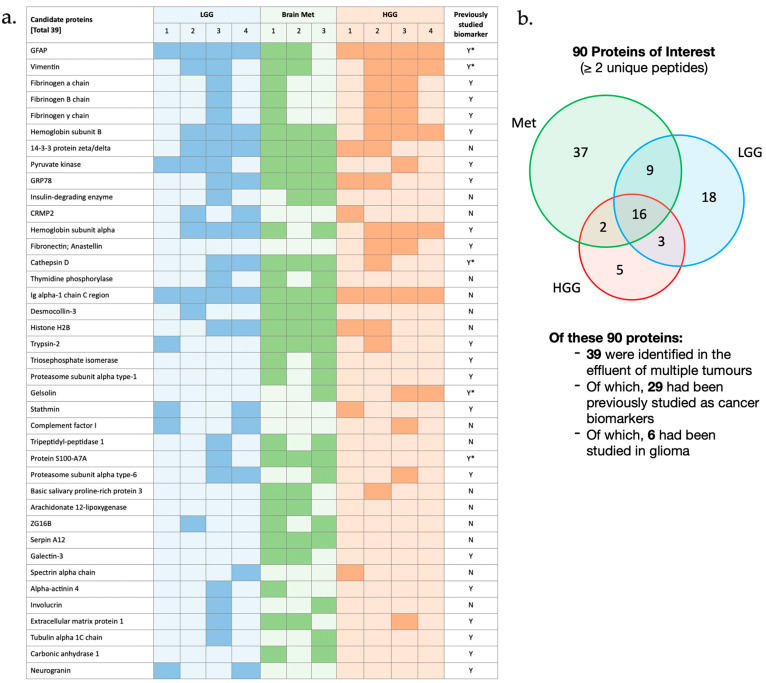
Summary of mass spectrometry data. (**a**) Table focuses on the 39 proteins that were identified in the effluent of more than one tumour. The darkly shaded boxes indicate the tumour effluent in which each protein was detected. The final column indicates whether the protein has previously been studied as a diagnostic cancer biomarker (Y = Yes, N = No, Y* = previously studied as a glioma biomarker); (**b**) representation of all 90 tumour tissue-derived proteins of interest. The Venn diagram demonstrates differential detection of proteins in the effluent of each tumour type.

A total of 299 proteins were detected in the tissue effluent using mass spectrometry. Of these, 197 proteins were identified by at least two unique peptides and therefore chosen for further evaluation [36]. Proteins that were identified in both the tissue effluent and the control media were excluded, leaving a total of 90 tissue-derived proteins of interest. The differential detection of proteins across the GBM (high grade glioma; HGG), LGG, and brain metastasis tumour types is visualised in Figure 3. It was noted that 16 of the proteins were found in all three tumour types, while 60 were found only in a single tumour type.

Of the 90 initial proteins of interest, 39 were found in the effluent of more than one tumour and were therefore prioritised as candidates for further evaluation. To establish the utility of the model in isolating and identifying novel biomarkers, the literature was reviewed to determine whether any of the candidate proteins had previously been validated as serum cancer biomarkers. The majority (79%) of the 39 proteins had previously been shown to exhibit altered expression in cancerous tissues or cell lines. Notably, 29 (74%) of the proteins had been investigated as diagnostic or prognostic biomarkers in cancer with positive results, and of these, six (15%) had been specifically studied in glioma.

### 3.5. Quantification of Biomarker Release Using ELISA

**Figure 4:**
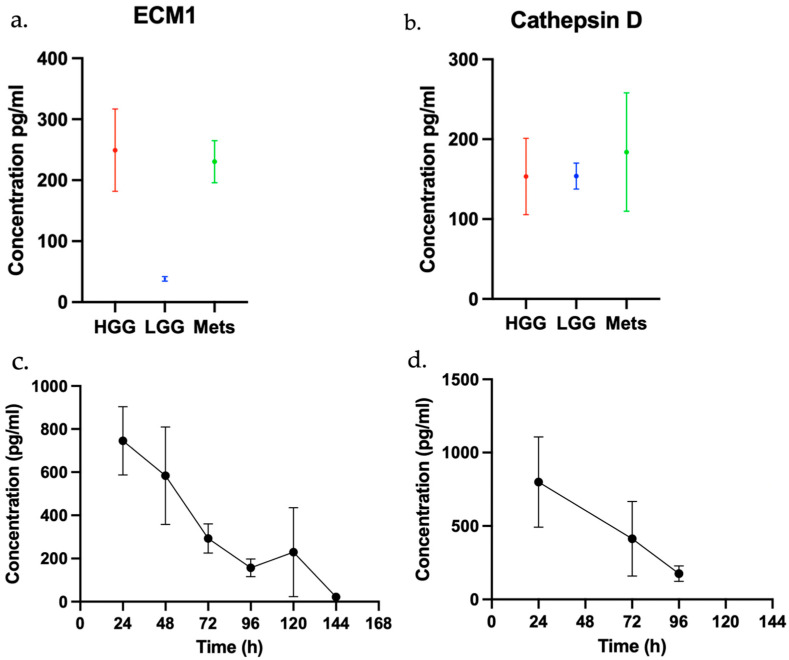
Concentration of ECM1 and cathepsin D in the tumour tissue effluent as determined by ELISA: (**a**) average ECM1 concentration in the tissue effluent for GBM (high grade glioma; HGG) (72 h, n = 12) and LGG (96 h, n = 7) and Mets (96 h, n = 2); (**b**) average cathepsin D concentration in the tissue effluent for GBM (72 h, n = 7) and LGG (96 h, n = 3) and Mets (96 h, n = 8); (**c**) average ECM1 concentration in the tissue effluent between 24 and 144 h, pooled for all tumour types (n = 9); (**d**) average cathepsin D concentration in the tissue effluent between 24 and 96 h, pooled for all tumour types (n = 7).

Two of the candidate biomarkers were selected for quantitative characterisation within the tissue effluent. Cathepsin D and ECM1 were chosen on the basis that they were each found in the effluent of all three tumour types. In addition, both had been reported in the literature as having potential utility as diagnostic cancer biomarkers [37,38]. The concentration of ECM1 within the tissue effluent differed significantly between tumour types (F(2, 22) = 3.682, *p* = 0.0418). The effluent concentration of ECM1 in the GBM (high grade glioma; HGG) samples (249.3 ± 67.1 pg/mL) was significantly higher (*p* = 0.0407) than that of the LGG samples (37.7 ± 4.23 pg/mL); however no significant difference (*p* = 0.9731) was noted between the GBM and metastasis samples (230.4 ± 34.4 pg/mL). In contrast, there was no significant difference (F(2,15) = 0.07612, *p* = 0.9271) in concentration of cathepsin D between the GBM samples (153.4 ± 47.8 pg/mL), LGG samples (153.9 ± 16.23 pg/mL), and metastasis samples (183.9 ± 74.4 pg/mL). The pooled concentration of proteins across all tumour types was plotted over time. This reveals a trend of reducing concentration of both cathepsin D and ECM1 throughout the period of ex vivo perfusion (Figure 4).

## 4. Discussion

There is a need for non-invasive diagnostic tools to support current radiological and histopathological methods in diagnosing brain tumours. While plasma biomarkers are routinely used in the diagnosis and monitoring of cancers such as prostate, pancreas, and ovary, no such markers have been validated for clinical use in brain tumours, despite promising preclinical findings. Here, we present a novel approach to brain tumour biomarker discovery that circumvents some of the limitations associated with standard methodologies that rely on proteomic characterisation of patient plasma and cerebrospinal fluid.

In the present study, we maintained 55 tumour samples from 11 different brain tumours on a miniaturised tissue perfusion device for a period of 7 days (168 h). This approach to ex vivo tissue maintenance has previously been demonstrated successfully with GBM [10]; however, to our knowledge this is the first instance in which low-grade glioma and brain metastasis tissue has been maintained in a similar manner. We used LDH cytotoxicity assays and histology to demonstrate that all tumour types studied can retain viability over a 7-day period of ex vivo perfusion. While there was some disturbance to the tissue architecture over that period, cellularity was maintained in the majority of samples and pathological diagnosis of the tumour was possible by a histopathologist. Tumour-specific cytomorphological features remained readily appreciable amongst the GBM and colorectal metastasis samples.

Using RP-cLC-MS, we identified 90 tissue-derived proteins in the tumour effluent, of which 39 were prioritised for further evaluation on the basis that they were present in the effluent of multiple tumours. A review of the literature revealed that a significant majority of these proteins had previously been implicated in cancer biology, and 29 had been studied as potential diagnostic or prognostic cancer biomarkers. One such example is GFAP, which was identified in the effluent of all tumour types in this study. GFAP is a cytoskeletal intermediate filament protein primarily expressed in astrocytes and neural stem cells within the central nervous system. Plasma GFAP levels have been shown to correlate with clinical severity and extent of intracranial injury across various brain pathologies including traumatic brain injury and stroke [39]. GFAP has therefore garnered significant attention as a potential diagnostic biomarker for aggressive brain tumours. A meta-analysis of 28 studies reported that 62.7% of high-grade glioma patients had detectable serum GFAP levels, compared to 12.7% of healthy controls, and a similar trend was also recognised for GFAP levels in CSF [7]. Despite the established association between plasma GFAP and brain tumours, routine GFAP testing does not yet have a role in clinical practice, primarily due to its relatively low diagnostic sensitivity and the limited data on its specificity in distinguishing between high- and low-grade glioma subtypes.

We selected two proteins identified by mass spectrometry—cathepsin D and ECM1—for further evaluation by ELISA to investigate the differential release into the effluent media between time points and tumour types. Cathepsin D is a lysosomal endopeptidase secreted from cancer cells; it acts as a mitogen on both cancer and stromal cells, and its tissue expression has been shown to correlate with increasing grade of glioma [40]. Fukuda et al. (2005) [41] showed that plasma cathepsin D level was an independent predictor of overall survival in patients with GBM. In our study, we did not find a significant difference in the concentration of cathepsin D in the tissue effluent between tumour subtypes by as measured by ELISA [41]. While this appears to contradict the findings of Fukuda et al., it is noted that five of the 11 patients with GBM in their cohort did not show raised serum levels of cathepsin D. It is therefore possible that our smaller cohort failed to capture the subset of high cathepsin D GBM patients identified in their study. Extracellular matrix protein 1 is a secreted glycoprotein with recognised roles in cell proliferation, angiogenesis and differentiation. Elevated ECM1 expression has been reported in several tumour types, including breast invasive ductal carcinoma and oesophageal squamous cell carcinoma, and has been associated with increased metastatic potential [42]. In GBM, silencing of ECM1 expression has been shown to inhibit cell proliferation, migration, and invasion in vitro [43]. Although there is no previous research regarding the use of ECM1 as a biomarker for glioma, Wang et al. (2017) demonstrated significantly elevated expression of ECM1 in the plasma of patients with oesophageal squamous cell carcinoma (n = 28) compared to healthy controls (n = 28) [37].

In the present study, we demonstrated by ELISA that the concentration of ECM1 within the effluent media of the GBM tumour samples was significantly higher than that of the LGG samples. This finding suggests the potential utility of ECM1 as a plasma marker for distinguishing between high- and low-grade glioma subtypes. While glioma grade can often be predicted based on neuroradiological features, a substantial proportion of high-grade gliomas lack the classical distinguishing characteristic of contrast enhancement [44]. Misclassifying a non-enhancing high-grade glioma as a low-grade tumour can lead to delays in biopsy, diagnosis, and treatment. The identification and validation of a serological marker that can help differentiate non-enhancing high- and low-grade gliomas has the potential to improve patient treatment.

In addition to ECM1, we identified numerous proteins in the tumour tissue effluent that have not previously been reported in the context of brain tumour biomarker research. Some of these proteins were detected exclusively in specific tumour subtypes by mass spectrometry. For example, Serpin A12—a serine protease inhibitor previously implicated as a marker of insulin resistance and diabetes [45]—was detected in the effluent of all brain metastasis samples but was absent from glioma samples. In contrast, dihydropyrimidinase-related protein 2 (CRMP2)—a tubulin-binding protein involved in neuronal development [46]—was present in the effluent of multiple primary brain tumours and notably absent from all brain metastasis samples. We recommend further validation of these findings in a larger cohort, along with quantification by ELISA to ensure reproducibility. Once a robust panel of candidate diagnostic biomarkers is proposed based on proteins identified in the tumour effluent, subsequent steps will include assessing differential levels of these proteins in patient serum. Biomarkers consistently elevated in patients with brain tumours may represent clinically useful biomarkers, with potential to form the basis of simple blood-based diagnostic assays to assist tumour detection and diagnosis. Given the complexity of tumour biology, it is likely that a panel of multiple protein markers will be required to achieve the sensitivity and specificity thresholds necessary for clinical applications due to the high level of inter-patient variation.

The miniaturised tissue perfusion model described in this study provides an inexpensive and efficient platform for investigating brain tumour biology. This system allows for the simultaneous maintenance of multiple samples from the same tumour, helping to account for intra-tumour heterogeneity and increasing sensitivity to capture proteins that may not be uniformly expressed across the tumour cell population. Furthermore, this system permits the detection of proteins derived directly from the tumour microenvironment rather than those released by all the other tissues in reaction to brain tumourigenesis.

Our current study is limited to a degree by a relatively small sample number for each tumour type. It is likely that the tumour markers identified in our cohort are not fully representative of those expressed by brain tumours generally, although it is reassuring that several of the proteins we identified have been previously studied as tumour markers. We also observed varying degrees of tissue necrosis and disruption of tissue architecture between samples following ex vivo perfusion. While necrosis is an expected histological feature of high-grade tumours, it is not typical of low-grade tumours. The necrosis of low-grade tissue during perfusion may result in the release of proteins into the effluent in a manner that is not representative of in vivo tumour biology. It is therefore particularly important that future research can demonstrate the presence of these markers in the serum or CSF of patient with low-grade brain tumours. Surgical excision and subsequent micro-dissection can affect tissue integrity and morphology, optimal sample processing is therefore essential to minimise this. The effects of ex vivo perfusion on brain tumour tissue are yet to be fully elucidated; it would be informative to establish a clearer understanding of how microenvironmental components such as microglia are affected by perfusion. We also recognise the possibility that the detection of extracellular matrix-associated proteins, such as ECM1, in the tumour effluent may reflect passive degradation of the tissue architecture during ex vivo perfusion rather than active secretion by tumour cells. However, Santasusagna et al. (2018) demonstrated increased levels of exosomal ECM1 in the plasma of patients with colon cancer compared to controls, suggesting a potential mechanism of active exosomal secretion of ECM1 by tumour cells, although a similar phenomenon is yet to be demonstrated in patients with glioma [47].

To improve the efficiency of our methodology, it is likely that samples need not be maintained on-chip for the full 168-hour duration. Cell death, as indicated by stabilisation of LDH release, plateaus by 96 h; therefore, effluent collection and analysis at this earlier time point may be sufficient for biomarker detection. We also demonstrated, by ELISA, that the concentration of effluent proteins cathepsin D and ECM1 show a decreasing trend during the period of perfusion, suggesting that sampling at earlier time points may increase sensitivity to the detection of such proteins. Research by Moursi et al. (2022), using a similar model to maintain human GBM tissue ex vivo, demonstrated that the concentration of tumour-derived proteins in the effluent do not universally decrease over time [48]. They reported several cytokines that showed persistently high or increasing concentrations over the period of perfusion.

If the model is to be used to assess responses to anti-cancer treatments, seeking to improve tissue viability over a longer period—shown here to be feasible—may be advantageous. By integrating the paradigms of treatment and biomarker discovery, it may be possible to identify markers of treatment response. This could be particularly useful in the monitoring of patients with GBM, specifically in differentiating disease progression from pseudoprogression—a phenomenon in which the response of the tumour to treatment mimics the radiological appearances progression.

## 5. Conclusions

Here, we demonstrate for the first time the ability to maintain multiple brain tumour subtypes ex vivo for 168-h with the use of a miniaturised tissue perfusion device. These findings broaden the range of tumours for which this inexpensive and efficient model has been proven to be effective. With this platform, we performed an unbiased proteomic interrogation of the tumour effluent and identified several biomarkers that have previously demonstrated potential diagnostic utility in glioma. The use of mass spectrometry to interrogate ex vivo tumour tissue effluent in this manner represents a novel approach to brain tumour biomarker discovery, and the tumour-derived proteins that can be identified with this platform may have translational potential as candidates for blood-based diagnostic assays. We have reported multiple tumour-derived proteins—including ECM1, Serpin A12 and CRMP2—as promising candidates that warrant further research to establish their expression in the plasma or CSF of patients with gliomas and brain metastases.

## Figures and Tables

**Table 1:** Patient Characteristics.

Tumour Classification	Histological Diagnosis	WHO Grade	Primary or Recurrent	Age Range (years)	Sex	IDH1 Status	Pre-Operative Steroids	Prior Chemotherapy
LGG	Astrocytoma	2	Recurrent	30–40	F	Mutant	No	No
LGG	Astrocytoma	2 ^1^	Primary	20–30	M	Mutant	Yes	No
LGG	Astrocytoma	2	Primary	30–40	M	Mutant	No	No
LGG	Oligodendroglioma	2 ^2^	Primary	20–30	M	Mutant	No	No
GBM	Glioblastoma	4	Primary	40–50	M	Wild type	Yes	No
GBM	Glioblastoma	4	Primary	50–60	F	Wild type	Yes	No
GBM	Glioblastoma	4	Primary	40–50	F	Wild type	Yes	No
GBM	Glioblastoma	4	Primary	50–60	M	Wild type	Yes	No
Brain metastasis	Metastatic lung adenocarcinoma	-	-	70–80	F	-	No	No
Brain metastasis	Metastatic breast carcinoma	-	-	70–80	F	-	No	Yes ^3^
Brain metastasis	Metastatic colorectal adenocarcinoma	-	-	60–70	M	-	Yes	Yes ^4^

^1^ WHO CNS grade 2 with <10% area of progression to 3. ^2^ Predominantly WHO CNS grade 2 with focal areas of increased mitotic activity. ^3^ Prior treatment with letrozole and anti-HER2 monoclonal antibody therapy. ^4^ Prior treatment with oxaliplatin, irinotecan, fluorouracil, and leucovorin (FOLFIRINOX). LGG = low grade glioma, GBM = glioblastoma, WHO = World Health Organisation.

## Data Availability

The raw data supporting the conclusions of this article will be made available by the authors on request.

## Abbreviations

The following abbreviations are used in this manuscript:

LGG	Low grade glioma
HGG	High grade glioma
GBM	Glioblastoma
CSF	Cerebrospinal fluid
CNS	Central nervous system
ECM1	Extracellular matrix protein 1
GFAP	Glial fibrillary acidic protein
LDH	Lactate dehydrogenase
RP-cLC-MS	Reversed-phase capillary liquid chromatography-mass spectrometry
IDH	Isocitrate dehydrogenase
ELISA	Enzyme-linked immunosorbent assay
CRMP2	Collapsin response mediator protein 2
NHS	National Health Service
DNA	Deoxyribonucleic acid
ctDNA	Circulating tumour DNA
RNA	Ribonucleic acid
EV	Extracellular vesicle
ANOVA	Analysis of variance
SEM	Standard error of the mean
MRI	Magnetic resonance imaging

## Appendix A

**Table A1:** Media Composition.

Media	Components	Tissue
NB media	Neurobasal medium (Thermo Fisher Scientific, MA, USA) B-27 supplement (Thermo Fisher Scientific, MA, USA) N-2 supplement (Thermo Fisher Scientific, MA, USA) Recombinant human EGF (Bio-Techne, MN, USA) Recombinant human beta-FGF (Thermo Fisher Scientific, MA, USA) Penicillin (0.1 U/mL) (Merck KGaA, Darmstadt, Germany) Streptomycin (0.1 g/mL) (Merck KGaA, Darmstadt, Germany)	Low grade glioma High grade glioma
Adenocarcinoma media	RPMI 1640 (Thermo Fisher Scientific, MA, USA) Penicillin (0.1 U/mL) Streptomycin (0.1 g/mL)	Metastatic colorectal adenocarcinoma Metastatic lung adenocarcinoma
Breast media	DMEM F12 (Thermo Fisher Scientific, MA, USA) Penicillin (0.1 U/mL) Streptomycin (0.1 g/mL)	Metastatic breast adenocarcinoma
